# 868. Contact Points of Electrical Injury: Disproportionate Outcomes by Ethnicity, Region, and Payor

**DOI:** 10.1093/jbcr/irag033.345

**Published:** 2026-04-06

**Authors:** Nathan H Brown, Mario Rivera, Claudia Leonardi

**Affiliations:** University Medical Center, Louisiana State University Health Sciences Center, New Orleans, New Orleans, LA; The Burn Center at University Medical Center New Orleans, New Orleans, LA; Louisiana State University Health Sciences Center, New Orleans, LA

## Abstract

**Introduction:**

Electrical injuries are relatively rare in the United States, accounting for approximately 3% of all burn center admissions. Nonetheless, despite their relative infrequency, they remain one of the leading causes of workplace fatalities and injuries in the United States.

The objective of this study is to analyze data from the American Burn Association’s (ABA) burn care quality platform (BCQP) registry to compare electrical and non-electrical burn injuries by demographic and clinical characteristics, patient outcomes, and regional patterns, with the goal of identifying disparities and guiding targeted prevention and care strategies.

**Methods:**

In this retrospective study, we examined data from the BCQP Registry. In our study, we analyzed all admitted adult cases assessed by burn services between 2020 through 2022 for treatment of a burn or other soft tissue wound.

Categorical variables were compared using chi-square tests, while continuous variables, summarized as medians with interquartile ranges (IQRs), were compared using the non-parametric Wilcoxon rank-sum test. Statistical significance was defined as a *p*-value less than 0.05.

**Results:**

Overall individuals who experienced a primarily electrical burn injury were approximately 36 years old, overwhelmingly male (89.0%), non-Hispanic (70.9%), White (66.3%), and injured on the job (55.3%).

Patients with electrical burn injuries had both shorter median hospital stay (2 vs. 4 days, *p*<.0001) and median ICU stay (3 vs. 4 days, *p*<.0001). Mortality was significantly lower in the electrical burn group (2.1% vs. 3.5%, *p*=.0002).

The southern region is physically vast and populous, but the rate of electrical injuries per 1000,000 people per year is also higher in the Southern region than all other regions: Southern (6.3), Midwest (4.8), Western (3.2), Eastern Great Lakes (3.1), and Northeast (2.7).

**Conclusions:**

First, in the present sample, individuals with an electrical injury were more likely to be Hispanic/Latino and reside in the Southern region. Second, the payor is also significantly different among those burned by electrical injuries. Third, we found that electrical injuries lead to relatively better overall medical outcomes. Finally, among patients with electrical injuries, the majority (51.7%) were treated in the Southern region and the rate of electrical injuries is higher in the Southern region than all other ABA regions.

**Applicability of Research to Practice:**

The following recommendations may be first steps toward prevention. First, it is likely that language appropriate safety training would reduce workplace electrical injuries. Second, workers would be better protected with an increase in laws regulating safety training and safe work practices, as well as a greater emphasis on electrical hazard awareness. Third, there is some evidence to suggest that targeted and peer-led health and safety programs result in fewer injuries and greater use of personal protective equipment.

**Funding for the study:**

N/A.

